# FruitNet: Indian fruits image dataset with quality for machine learning applications

**DOI:** 10.1016/j.dib.2021.107686

**Published:** 2021-12-07

**Authors:** Vishal Meshram, Kailas Patil

**Affiliations:** Vishwakarma University, India

**Keywords:** Convolutional neural network, Computer vision, Deep learning, Fruit classification, Fruit detection, Fruit image dataset, Machine learning

## Abstract

Fast and precise fruit classification or recognition as per quality parameter is the unmet need of agriculture business. This is an open research problem, which always attracts researchers. Machine learning and deep learning techniques have shown very promising results for the classification and object detection problems. Neat and clean dataset is the elementary requirement to build accurate and robust machine learning models for the real-time environment. With this objective we have created an image dataset of Indian fruits with quality parameter which are highly consumed or exported. Accordingly, we have considered six fruits namely apple, banana, guava, lime, orange, and pomegranate to create a dataset. The dataset is divided into three folders (1) Good quality fruits (2) Bad quality fruits, and (3) Mixed quality fruits each consists of six fruits subfolders. Total 19,500+ images in the processed format are available in the dataset. We strongly believe that the proposed dataset is very helpful for training, testing and validation of fruit classification or reorganization machine leaning model.

**Specifications Table**

**Table:** 

Subject	Machine learning, agriculture science, horticulture
Specific subject area	Fruits image dataset with quality classification (good, bad, and mixed)
Type of data	Indian fruits images
How data were acquired	Fruits images were using high resolution mobile phone camera in the natural and artificial light conditions with different backgrounds.
Data format	Raw
Parameters for data collection	The fruit dataset images are .jpg images of 256 × 256 dimension and resolution is 72 dpi.
Description of data collection	The fruits images were collected using high resolution mobile phones rear camera. The original .jpg images of fruits are of dimensions 3024 × 3024. These images are resized to 256 × 256 dimensions. The dataset is categorized into 3 subfolders Good Quality Fruits, Bad Quality Fruits, and Mixed Quality Fruits. Further each folder contain six fruits classes namely Apple, Banana, Guava, Lime, Orange, Pomegranate. The images were taken at the different backgrounds and in different lighting conditions. The proposed dataset can be used for training, testing and validation of fruit classification or reorganization model.
Data source location	**VISHWAKARMA UNIVERSITY** Survey No. 2, 3, 4 Laxmi Nagar, Kondhwa Budruk, Pune - 411 048. Maharashtra, India. Latitude and longitude: 18.4603° N, 73.8836° E **HUBTOWN COUNTRYWOODS SOCIETY** Tilekar Nagar, Kondhwa Budruk, Pune - 411 048. Maharashtra, India. Latitude and longitude: 18.442866 ° N, 73.884894° E
Data accessibility	Repository name: FruitNet: Indian Fruits Dataset with quality (Good, Bad & Mixed quality) Data identification number(doi): 10.17632/b6fftwbr2v.1 Direct URL to data: https://data.mendeley.com/datasets/b6fftwbr2v/1

**Value of the Data**

• The dataset is comprehensive which consist of 19500+ high-quality images of six different classes.
• The dataset consist of good quality, bad quality, and mixed quality fruit images.
• To the best of our knowledge this is the first open access dataset of indian fruits consistes of good, bad and mixed quality fruits.
• This dataset is useful to build applications of fruit classification and detection with quality.
• The dataset will be useful for training, testing and validation of fruit classification or reorganization model.
• The dataset is useful to build fruit classification with quality applications which are beneficial for farmers, agriculture industries, wholesalers, hawkers, and customers, and fruit export companies.

## 1 Data Description

**Fig. 1:**
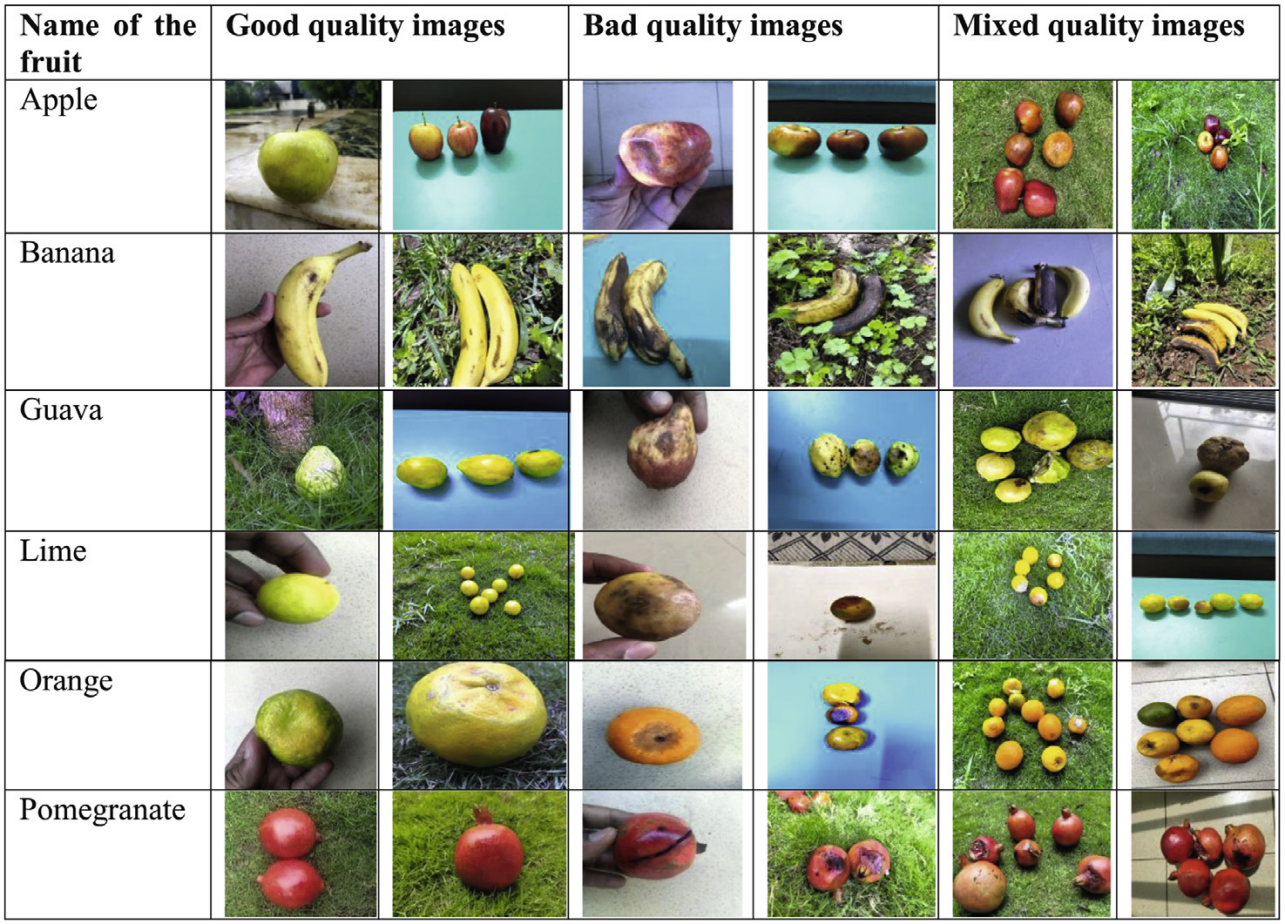
Partial images of the dataset.

The profit percentage share of fruit market is substantial with respect to the total agriculture output [1], [2], [3]. In the agro-industry fast and accurate fruit classification is the highest need. The fruits can be classified into different classes as per their external features like shape, size and color using some computer vision and deep learning techniques [4], [5], [6], [7], [8]. The FruitNet dataset was created to include Indian fruits along with its quality parameters for those which are highly consumed or exported as per [9]. It consists of six classes of Indian fruits namely apple, banana, guava, lime, orange, and pomegranate. They further categorized into good quality, bad quality, and mixed quality. The fruit images were taken with different background, in different light conditions in indoor and outdoor environment. The Fig. 1 shows the sample images in the dataset consisting of images taken in various environments.

## 2 Experimental Design, Materials and Methods

### 2.1 Experimental design

**Fig. 2:**
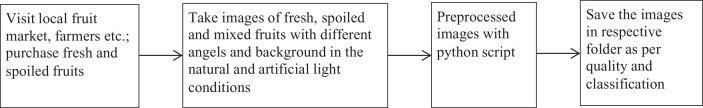
Fruits data acquisition process.

The image data acquisition process is shown in Fig. 2. The fruit images were acquired using three different make of camera's i.e. iPhone6 (Apple), ZUK (Z2 Plus), and Realme (Realme 5 Pro) mobile's high resolution rear camera. In all 19500+ images were captured using camera and then were segregated and saved in respective folders as per their quality and classification.

The data acquisition process steps are shown in Table 1. The fruit images are captured in the natural and artificial lighting conditions with different angles and background in months of July to October. Images pre-processing is done using python script. In the pre-processing we changed the dimensions to 256 × 256 which is standard resolution required to build object classification or object detection model.

**Table 1:** Data acquisition steps.

Sr. No.	Step	Duration	Activity
1.	Data Gathering	July to October	Daily captured the fruits images in the natural and artificial light with different angles and background.

2.	Pre-processing and creating dataset	November	Run the python script to pre-process the images (convert all images in 256 × 256 resolution) and save the images into respective folders as per their quality and classification (i.e. bad, good and mixed)

### 2.2 Materials or specification of image acquisition system

The fruit images are captured using Apple iphone6 with rear camera of 8 megapixels, Z2 plus with rear camera of 13 megapixel, and realme 5 pro with rear camera of 48 megapixels. All dataset images of original size 3024 × 3024 were resized to 256 × 256 dimensions using a python script. The images are in .jpg images. The images acquired in variety of environmental conditions such as different light conditions, different background, and from different angles.

After capturing the images were organized as Bad quality, Good quality, and Mixed quality folders. Further each quality folder has six different folders of fruit classes i.e. apple, banana, guava, lime, orange, and pomegranate, respectively. The specifications of devices used for image acquisition and acquired images specifications are shown in Tables 2 and 3, respectively.

**Table 2:** Specification of image acquisition device.

Sr. No.	Camera Particulars	Details		
1	Camera maker	Apple	ZUK	Realme
2	Camera Model	iPhone 6	Z2 Plus	Realme 5 Pro
3	F-stop	f/2.2	f/2.2	f/1.8
4	Exposure time	1/25 s	1/214 s	1/33 s
5	ISO Speed	ISO-250	ISO-100	ISO-1120
6	Exposure bias	0 step	0 step	0 step
7	Focal length	4 mm	4 mm	5 mm
8	metering mode	Pattern	Centered Weighted Average	Unknown
9	Flash mode	No flash	No flash	No flash
10	35mm focal length	29	29	0

**Table 3:** Specification of images.

		Details as per Fruit classes		
Sr. No.	Particulars	Bad Fruit	Good Fruit	Mixed Fruit
1	Dimension	256 × 256	256 × 256	256 × 192
2	Width	256 pixels	256 pixels	256 pixels
3	Height	256 pixels	256 pixels	192 pixels
4	Horizontal Resolution	72 dpi	96 dpi	72 dpi
5	Vertical Resolution	72 dpi	96 dpi	72 dpi
6	Bit Depth	24	24	24
7	Resolution unit	2	2	2
8	Color representation	sRGB	sRGB	Uncalibrated

### 2.3 Method

All fruit images are acquired using three mobile make with a high resolution rear camera in different angles and different backgrounds. The orignal images of size 3024 × 3024 were resized to 256 × 256 using a python script. Table 4 describes the classes, number of image taken and the environments in which images are taken.

**Table 4:** FruitNet dataset details.

Quality classes	Fruit classes Considered	Image Taken in which Direction	Image Taken in different Backgrounds	No. of Images of each denomination	Total No. of Images
Bad quality	apple, banana, guava, lime, orange, pomegranate	Front Direction, Top View, Backward Direction, Bottom View, Direction Rotated 180 degrees,	Dark color, grass, light color, ground, multicolor	apple - 1141 banana - 1087 guava - 1129 lime - 1085 orange - 1159 pomegranate - 1187	6778

Good quality	apple, banana, guava, lime, orange, pomegranate	Front Direction, Top View, Backward Direction, Bottom View, Direction Rotated 180 degrees,	Dark color, grass, light color, ground, multicolor	apple - 1149 banana - 1113 guava -1152 lime -1094 orange - 1216 pomegranate - 5940	11664

Mixed quality	apple, banana, guava, lime, orange, pomegranate	Front Direction, Top View, Backward Direction, Bottom View, Direction Rotated 180 degrees,	Dark color, grass, light color, ground, multicolor	apple – 113 banana – 285 guava – 148 lime – 278 orange – 125 pomegranate - 125	1074

**Total Number of Images in the Dataset**					**19526**

## Ethics Statement

There is no funding for the present effort. There is no conflict of interest. The data is available in public domain.

## CRediT authorship contribution statement

**Vishal Meshram:** Methodology, Software, Validation, Writing – original draft. **Kailas Patil:** Conceptualization, Supervision, Writing – review & editing.

## Declaration of Competing Interest

The authors declare that they have no known competing financial interests or personal relationships that could have appeared to influence the work reported in this paper.
